# Supplementary material for: The Role of Family Health in Mediating the Association between Smartphone Use and Health Risk Behaviors among Chinese Adolescent Students: A National Cross-Sectional Study
Source: Int J Environ Res Public Health. 2022 Oct 17;19(20):13378. doi: 10.3390/ijerph192013378 (PMC9603663; doi:10.3390/ijerph192013378)
Supplement: Supplementary file 1 [file ijerph-19-13378-s001.zip › ijerph-1953922-supplementary.pdf]

Table S1. Family Health Scale Short-form evaluation dimensions

Dimension	Items
**Family Social and Emotional Health Processes**	In my family, we support each other.
	In my family, I feel safe in my family relationships.
	In my family, we stay hopeful even in difficult times.
**Family Healthy Lifestyle**	In my family, we help each other in seeking health care services when needed (such as making doctor's appointments).
	In my family, we help each other make healthy changes.
**Family Health Resources**	In my family, we do not trust doctors and other health professionals.
	In the past 12 months, my family did not have enough money at the end of the month after bills were paid.
	In the past 12 months, my family did not have adequate housing.
**Family External Social Supports**	In my family, we have people outside of our family we can turn to when we have problems at school or work.
	In my family, if we needed financial help, we have people outside of our family we could turn to for a loan (e.g., for $200).

Table S2. Adolescent Health Risk Behaviors Scale measure items

Measure	Items
**How often did you do any of the following in the past 30 days?**	Non-compliance walking behavior (eg.,using phone while walking)
	Swimming in unguarded water (swimming in rivers and reservoirs)
	Non-compliance walking behavior (eg. running the red light)
	Not wearing seatbelt while riding
	Eating unhygienic food (eg.,food that has been contaminated with bacteria and has undergone partial spoilage)
	leaving home
	Subjected to physical violence
**What was the age of first full exposure to addictive substances (tobacco, alcohol)?**	The age at which a cigarette was first smoked
	The age of first alcohol consumption
